# Modelling malaria treatment practices in Bangladesh using spatial statistics

**DOI:** 10.1186/1475-2875-11-63

**Published:** 2012-03-05

**Authors:** Ubydul Haque, Lauren M Scott, Masahiro Hashizume, Emily Fisher, Rashidul Haque, Taro Yamamoto, Gregory E Glass

**Affiliations:** 1 International Center for Diarrheal Disease Research Bangladesh, Mohakhali, Dhaka, Bangladesh; 2 Department of Mathematical Sciences and Technology, Norwegian University of Life Sciences, Ås, Norway; 3 Environmental System Research Institute (ESRI), California, USA; 4 Department of International Health, Institute of Tropical Medicine (NEKKEN) and the Global, Center of Excellence Programme, Nagasaki University, Nagasaki, Japan; 5 University of Bergen, Bergen, Norway; 6 Department of Molecular Microbiology and Immunology, John Hopkins Bloomberg School of Public Health, Baltimore, MD 21205, USA

**Keywords:** Malaria treatment, Malaria control, Bangladesh, Local Getis-Ord Gi statistic, Spatial regression, Hot-spot analysis, Geographically weighted regression (GWR), GIS

## Abstract

**Background:**

Malaria treatment-seeking practices vary worldwide and Bangladesh is no exception. Individuals from 88 villages in Rajasthali were asked about their treatment-seeking practices. A portion of these households preferred malaria treatment from the National Control Programme, but still a large number of households continued to use drug vendors and approximately one fourth of the individuals surveyed relied exclusively on non-control programme treatments. The risks of low-control programme usage include incomplete malaria treatment, possible misuse of anti-malarial drugs, and an increased potential for drug resistance.

**Methods:**

The spatial patterns of treatment-seeking practices were first examined using hot-spot analysis (Local Getis-Ord Gi statistic) and then modelled using regression. Ordinary least squares (OLS) regression identified key factors explaining more than 80% of the variation in control programme and vendor treatment preferences. Geographically weighted regression (GWR) was then used to assess where each factor was a strong predictor of treatment-seeking preferences.

**Results:**

Several factors including tribal affiliation, housing materials, household densities, education levels, and proximity to the regional urban centre, were found to be effective predictors of malaria treatment-seeking preferences. The predictive strength of each of these factors, however, varied across the study area. While education, for example, was a strong predictor in some villages, it was less important for predicting treatment-seeking outcomes in other villages.

**Conclusion:**

Understanding where each factor is a strong predictor of treatment-seeking outcomes may help in planning targeted interventions aimed at increasing control programme usage. Suggested strategies include providing additional training for the Building Resources across Communities (BRAC) health workers, implementing educational programmes, and addressing economic factors.

## Background

Malaria is the most widespread parasitic disease in the world today and a major health burden in many tropical and sub-tropical regions of Africa, the Americas, Eurasia, and Oceania. It is endemic in 106 countries, putting half of the world's population (3.3 billion people) at risk [1]. In 2009, an estimated 225 million cases of malaria worldwide accounted for approximately 781,000 deaths [2].

In Bangladesh, malaria is endemic in 13 of the 64 administrative districts. Over the last five years, Bangladesh has received more than $80 million USD from the Global Fund to support a National Malaria Control Programme (NMCP) integrating rapid diagnostic tests (RDTs), new drug regimes using artemisinin-based combination therapy (ACT), expanded distribution of long-lasting insecticide-treated nets (LLIN), re-treatment of insecticide-treated nets (ITN), and vector surveillance [3]. In addition, a total of 1,676 indigenous health workers were recruited in remote and hard-to-reach areas of Bangladesh to supplement government health workers. These indigenous health workers were to be available in their own communities to provide RDT diagnostic services and to administer appropriate treatment when indicated [3]. The Programme's overall target is the reduction of the malaria burden (morbidity and mortality) by 60% before the year 2015 (using the 2008 malaria incidence as a baseline). In addition, the Programme seeks to provide LLINs to all households in the three highest endemic districts, to diagnose and effectively treat 90% of all malaria cases using control programme resources, and to raise awareness in malaria endemic districts regarding treatment options and malaria prevention strategies [3].

The success of the NMCP in Bangladesh will be measured by its ability to meet the above objectives. Previous studies [4], however, indicate that individuals in local communities have not taken full advantage of the programme resources available to them. Two years after the initial implementation of the NMCP, the most common strategy for malaria-associated fever has been to obtain treatment from BRAC or from other government operated facilities (66%). However, 49% of the individuals in the villages surveyed still obtained some portion of their malaria treatment from local drug vendors [4].

Because malaria treatment-seeking practices differ around the world [5-21], no universal strategy can be developed to tackle the issue of malaria incidence and treatment. Efforts to tailor malaria control programmes to local needs, requires an understanding of the factors that influence individual treatment-seeking practices. In this paper, spatial pattern analysis techniques and spatial regression are used to illustrate where national control programme services are well-utilized and where they are under-utilized, to identify the factors contributing to alternative treatment-seeking preferences, and to assess how the predictive strength of those factors change across the study area. Understanding where each factor is a strong predictor of treatment-seeking preferences can inform the design of targeted interventions aimed at increasing control programme utilization. Given the results of the spatial analysis presented, a variety of possible intervention strategies are suggested.

## Methods

### Study area

**Figure 1:**
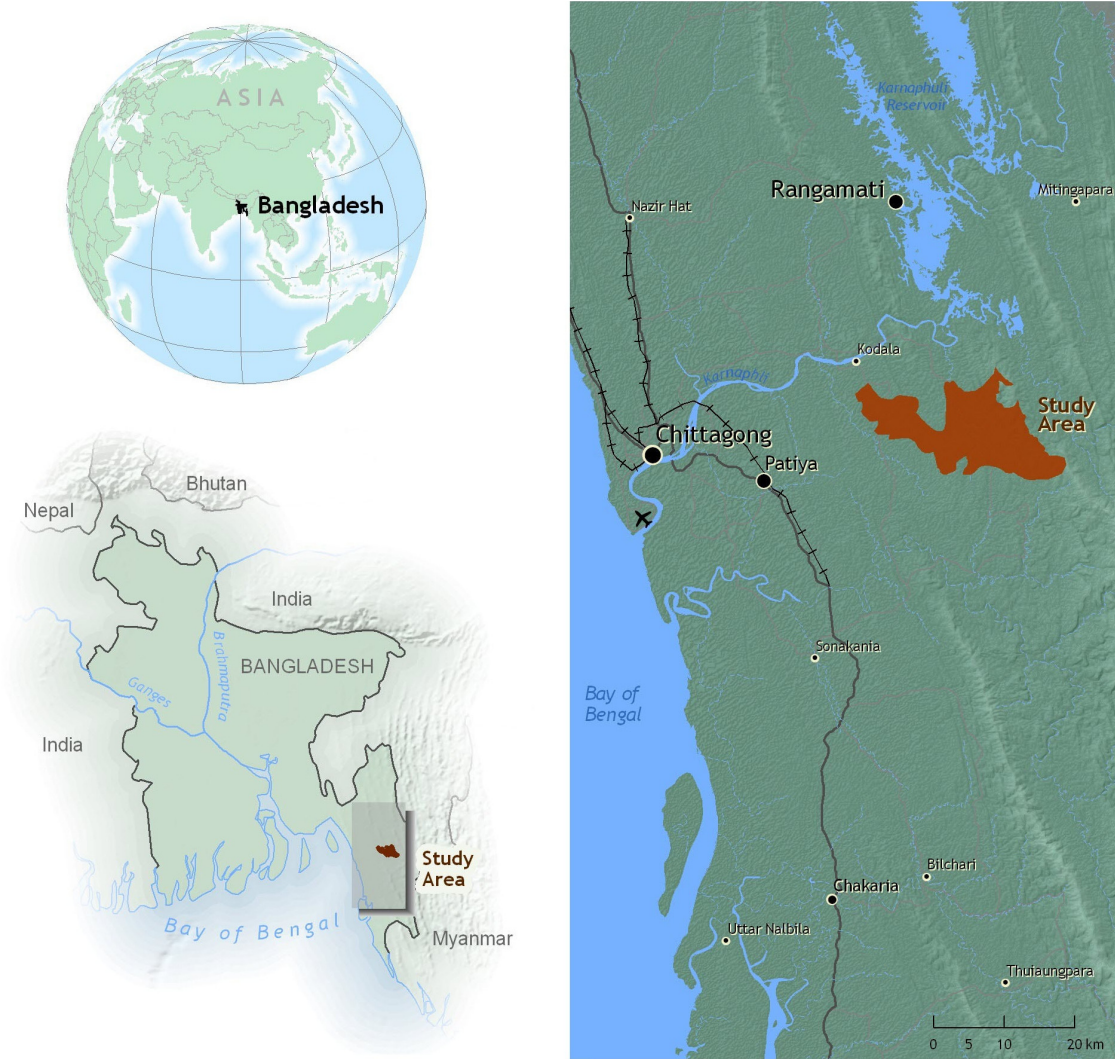
**Locator map**.

This study was carried out in the remote, forest-covered Chittagong Hill Tracts (CHT) of Rajasthali, Bangladesh (Figure 1), with a population of 24,097 [4]. All households and health facilities of Rajasthali were mapped using Global Positioning System (GPS) devices, distances from households to health facilities were computed, and a survey was administered asking about malaria-related treatment-seeking behaviour. In addition, 1,400 of the 5,322 households were screened for malaria using a rapid diagnostic test (Falci-vax). A full description of the data gathered, sampling techniques and logistics is provided in Haque *et al*, [4,22].

For this research, the sampled household data were aggregated into village totals and analysed within a Geographic Information System (GIS) environment [23]. Twenty-one villages with less than five household samples were excluded from the analysis. The remaining 88 villages were represented geographically by the mean centre [24] of their sampled households. Distance variables, such as distance to regional hospitals, were computed by summing, then averaging individual household distances.

### Hot-spot analysis

**Figure 2:**
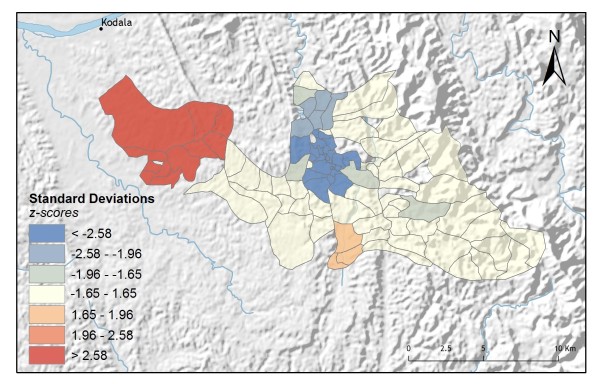
**Hot-spot analysis for vendor usage rates**.

**Figure 3:**
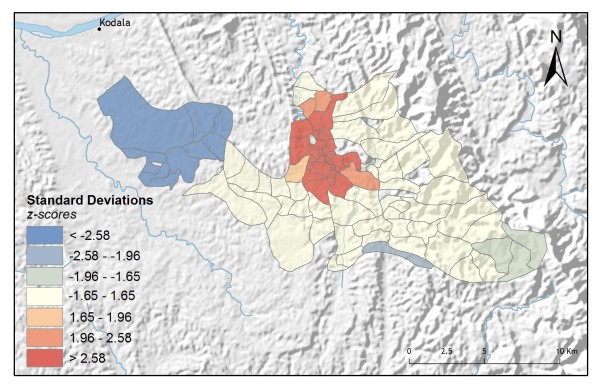
**Hot-spot analysis for control programme usage rates**.

The local Getis-Ord Gi statistic [25] provides a picture of malaria-related treatment-seeking variations in the study area. This statistic produces a hot-spot map (Figures 2 and 3), and was applied to both the vendor usage and the village control programme usage rates. The local Getis-Ord Gi statistic works by comparing the local mean rate (the rates for a village and its nearest neighbouring villages) to the global mean rate (the rates for all villages). It produces a *z-score *and *p-value *for each village, reflecting whether the differences between the local and global means are statistically significant or not. A statistically significant positive *z-score *indicates a hot spot for high rates where it is very unlikely that the spatial clustering of high values is the result of random spatial processes. Similarly, a statistically significant negative *z-score *for a village indicates a local spatial clustering of low rates (a cold spot) [25-27].

### Regression

With a picture of the spatial patterns of control programme and vendor use for villages within the study area, the next step was to model those treatment preferences using regression analysis. Results from ordinary least squares regression (OLS) can only be trusted, however, if they are derived from a properly specified model. A properly specified model is one that meets all of the requirements of the OLS method [28]: coefficients for model explanatory variables should be statistically significant and have the expected sign (+/-); explanatory variables must be free from multicollinearity; the model should not be biased (heteroscedasticity or non-stationarity); residuals must be normally distributed with a mean of zero; the model cannot be missing key explanatory variables; and residuals must be free from spatial autocorrelation [29].

A data-mining tool called Exploratory Regression was used to find a model that met all of the requirements of the OLS method. Exploratory Regression is similar to stepwise regression; instead of looking only for models with a high-adjusted R^2^, however, it identifies models that meet all of the requirements outlined above. The candidate variables used with the Exploratory Regression tool are described in Table 1. This method yielded two strong, properly-specified OLS models (Tables 2 and 3) [30]: one explaining the number of people using vendor services in each village (Adj R^2 ^= 0.90) and one explaining the number of people in each village using control programme services (Adj R^2 ^= 0.81). Both of these models were validated using bootstrapping and cross validation. For cross validation, the model was fit to a random sample of half the villages and validated against the remaining villages. For bootstrapping, 100 random samples (with replacement) were extracted and both models were tried. All of the explanatory variable coefficients were statistically significant in more than 95% of these random samples.

**Table 1:** Candidate explanatory variables

Variables	Values
Village size (total households)	8- 323 households

Household density within 2 km	41-1389 households

Malaria prevalence rate	0 - 75%

Bed-net usage and treatment	LLIN treatment rates, Number of bed-nets, Bed-net treatment rates, Proportion of family sleeping under a bed-net

Gender	0-100% for both males and females

Age	2.88 - 61

Altitude	26.67 - 265.54 meter

Forest	Forested portion of village 0.07 - 0.69

Accessibility to treatment	Distance to closest: BRAC facility 0.19 - 5.60 km, Hospital 0.55 - 7.37 km, Vendor services 0.18 - 7.12 km.

Tribal affiliation	Bengali, Marma, Tripura, Tonchonga, Khiang, Chakma

Housing materials	Material used for walls, roof, and floor

Employment	Proportion of village working in: Service/Business, small business, day labour occupations, agriculture, unemployment

Family size	3 - 7.25

Education	0 - 12 years, People with no schooling (uneducated)

Proximity and remoteness	Distance to roads: 7.28 - 4,087.5 m; Distance to urban centre; Interaction between distance to urban centre and village size (distance × size)

**Table 2:** Summary of OLS results for vendor use

Summary of OLS results: *Vendor use*								
**Variable**	**Coefficient**	**Std Errors**	**t-Statistic**	**Probability**	**Robust Std Errors**	**Robust t-Statistic**	**Robust Probability**	**VIF**

Intercept	-1.002419	0.617061	-1.624505	0.108021	0.526459	-1.90408	0.060325	--------------

Uneducated	0.547854	0.095300	5.748719	0.000000*	0.119265	4.59358	0.000017*	1.93

Straw Roofs	-0.624670	0.115432	-5.411565	0.000001*	0.142663	-4.37865	0.000036*	1.80

Distance × Size	0.000024	0.000001	20.656785	0.000000*	0.000001	19.83021	0.000000*	1.67

**OLS Diagnostics**								

Number of Observations:	88			Akaike's Information Criterion (AICc):				465.79

Multiple R-Squared:	0.9045			Adjusted R-Squared:				0.9011

Joint F-Statistic:	265.2562			Prob(> F), (3,84) degrees:				0.0000*

Joint Wald Statistic:	729.1270			Prob(> chi-squared), (3) degrees of freedom:				0.0000*

Koenker (BP) Statistic:	10.1478			Prob(> chi-squared), (3) degrees of freedom:				0.0174*

Jarque-Bera Statistic:	4.3285			Prob(> chi-squared), (2) degrees of freedom:				0.1148

**Table 3:** Summary of OLS results for control programme use

Summary of OLS results: *Control programme use*								
**Variable**	**Coefficient**	**Std Errors**	**t-Statistic**	**Probability**	**Robust Std Errors**	**Robust t-Statistic**	**Robust Probability**	**VIF**

Intercept	2.403869	1.164809	2.063745	0.042164*	1.346144	1.7574	0.077797	-------------

Household Density	0.005225	0.001231	4.244688	0.000059*	0.001370	3.38138	0.000267*	1.36

Bengali People	0.931071	0.057053	16.319406	0.000000*	0.092617	10.05295	0.000000*	2.34

Wooden Floors	0.873080	0.092385	9.450429	0.000000*	0.157137	5.55615	0.000000*	1.52

Distance × Size	-0.000016	0.000002	-8.175976	0.000000*	0.000003	-5.44154	0.000001*	2.09

**OLS Diagnostics**								

Number of Observations:	88			Akaike's Information Criterion (AICc):				532.97

Multiple R-Squared:	0.8189			Adjusted R-Squared:				0.8102

Joint F-Statistic:	93.8148			Prob(> F), (4,83) degrees:				0.0000*

Joint Wald Statistic:	171.0028			Prob(> chi-squared), (4) degrees of freedom:				0.0000*

Koenker (BP) Statistic:	33.0232			Prob(> chi-squared), (4) degrees of freedom:				0.0000*

Jarque-Bera Statistic:	0.2670			Prob(> chi-squared), (2) degrees of freedom:				0.8750

### Spatial regression

Geography matters: a variable that might be a strong predictor in one village may not necessarily be a strong predictor in another. To better understand this type of regional variation, Geographically Weighted Regression (GWR) was applied [31]. GWR is one of several spatial regression techniques used increasingly in geography and other disciplines. Rather than fitting a single linear regression equation to all of the data in the study area, GWR creates an equation for every feature (each village, in this case) and calibrates it using nearby features; closer features have a larger impact on calibration than features that are further away. Because each feature has its own equation, coefficients are allowed to vary over space [31]. Variations in tribal affiliations, education, economics, and access to treatment resources, for example, are likely to foster a variety of treatment preferences and community practices. Particularly useful outputs from GWR are maps of the coefficients associated with each explanatory variable. Where an explanatory variable has remediation implications, understanding where it is a strong predictor can provide guidelines for targeted interventions.

## Results

### Statistically significant hot spots

Individuals have multiple options for obtaining malaria treatment in Rajasthali and they commonly utilize more than one treatment strategy. For the 1,400 individuals surveyed, 42% indicated they would use vendor-provided services as well as other types of services, including control programme treatments; 73% indicated they would use control programme services as well as other types of services, including treatment from local vendors; 26% indicated they would seek treatment only from non-control programme options. These variations in treatment-seeking preferences were especially interesting when analysed spatially. Applying hot-spot analysis to vendor usage rates for each village (n = 88) revealed statistically significant (p < 0.01; z > 2.58) hot spots for the 10 western-most villages in the study area (Figure 2). In contrast, statistically significant hot spots for high control programme usage were located in the centre of the study area around the region's largest hospital and supplier of control programme services (Figure 3).

### Factors influencing control programme and vendor usage choices

**Figure 4:**
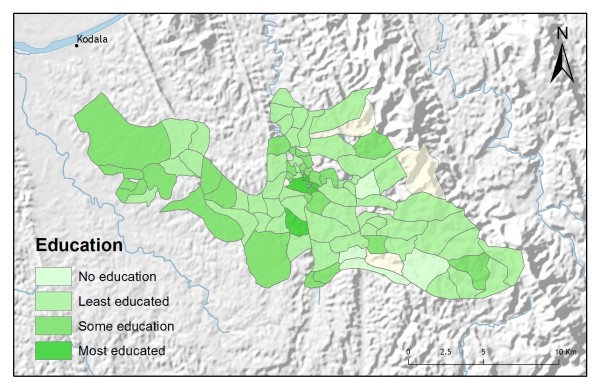
**Average years of education**.

**Figure 5:**
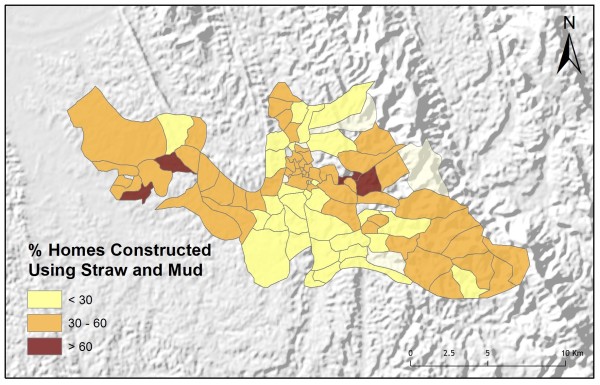
**Poorer villages have a larger proportion of homes constructed from straw and mud**.

**Figure 6:**
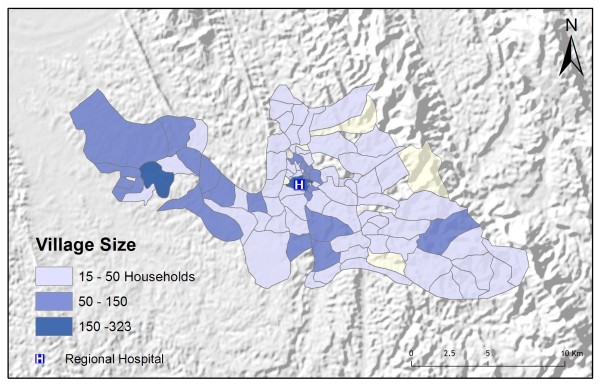
**Village size and distance from primary regional hospital**.

**Figure 7:**
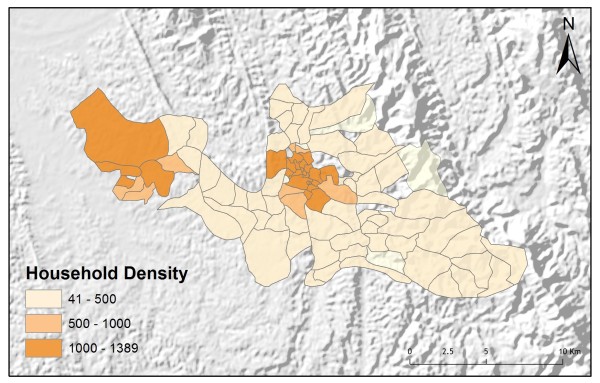
**Number of homes within two kilometers**.

**Figure 8:**
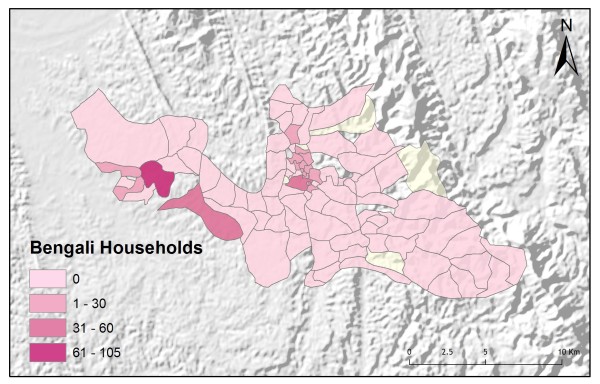
**Bengali population**.

**Figure 9:**
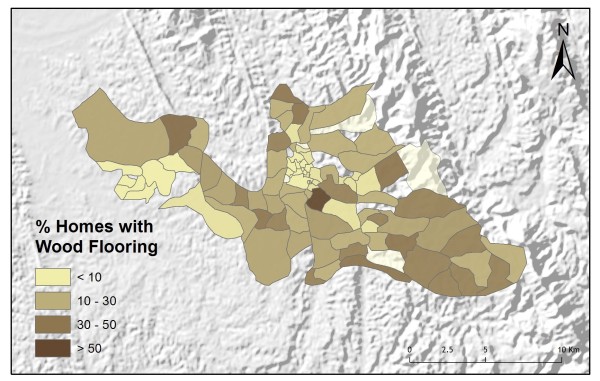
**Homes with floors constructed of wood**.

Table 2 shows the results from an OLS model of vendor usage. The dependent variable was the number of people in each village indicating they would use vendor services for their malaria treatment. This model explained more than 90% of the variation in vendor usage (Adj R^2 ^= 0.9011), and met all of the requirements of the OLS method [30]. The robust probabilities (based on White Standard Errors [32]) for the explanatory variable coefficients were statistically significant (p < 0.01), variance inflation factor (VIF) values were low (VIF < 7.5) indicating no problems with multicollinearity, the Joint Wald Statistic indicated overall model significance (p < 0.01), and the non-significant (p > 0.10) Jarque-Bera diagnostic indicated model residuals were normally distributed. The factors influencing vendor use include education (Figure 4), homes constructed from straw roof (poor economic status) (Figure 5), and an interaction term relating village size with distance to the regional urban centre where the largest regional hospital is located (Figure 6). Larger villages further from the regional hospital (the interaction term), with very little schooling (education), and better economic status (fewer homes constructed using straw roofing materials), were more likely to use vendor services. Factors influencing preference for control programme services, on the other hand, include household densities (Figure 7), tribal affiliation (Figure 8), housing construction materials (Figure 9), and, again, the interaction term relating village size to regional hospital proximity (Figure 6). This model (Table 3) explained 81% of the variation for people preferring control programme services (Adj R^2 ^= 0.8102), and indicated that the smaller, more compact, villages closest to the regional hospital, with wood flooring, and larger numbers of Bengali residents were more apt to take advantage of these services.

### Exploring non-stationarity

While OLS effectively identified the factors contributing to both control programme and vendor usage, OLS is a global model that assumes the relationship between each explanatory variable and the dependent variable is consistent (stationary) across the study area. In the case where relationships are non-stationary, model fit will improve by using GWR [31]. The adjusted R^2 ^value for vendor usage, for example, increased from 0.90 using OLS (Table 2) to 0.95 using GWR (Table 4). The corrected Akaike's Information Criterion (AICc) value from the OLS model (AICc = 465.79) was larger than the AICc value from the GWR model (AICc = 423.89). AICc is an effective way to compare models [33] and a drop of even three points indicates an important improvement in model fit [31]. Similarly, the adjusted R^2 ^value for control programme usage (Table 3) increased from 0.81 using OLS to 0.85 using GWR (Table 5), with a corresponding drop in AICc value from 532.97 with OLS to 515.66 with GWR.

**Table 4:** GWR model for vendor use count

Explanatory variables	Uneducated, straw roofs, distance × size
Residual squares	3691.58

Effective number	25.24

Sigma	2.34

Akaike's Information Criterion (AICc)	423.89

Multiple R-Squared	0.96

Adjusted R-Squared	0.95

**Table 5:** GWR model for malaria control programme count

Explanatory variables	Household density, Bengali people, wooden floors, distance × size
Residual squares	1309.63

Effective number	13.60

Sigma	4.20

Akaike's Information Criterion (AICc)	515.66

Multiple R-Squared	0.87

Adjusted R-Squared	0.85

**Figure 10:**
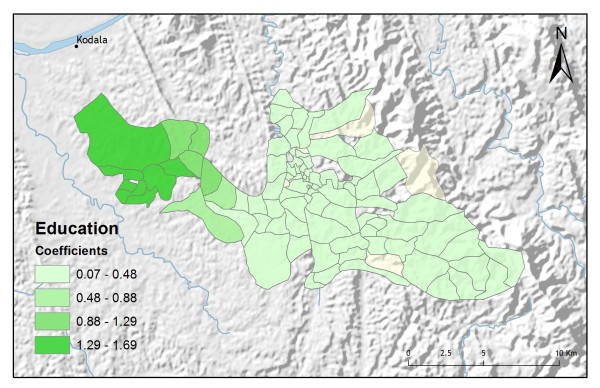
**Education GWR coefficients for predicting vendor service preference**.

**Figure 11:**
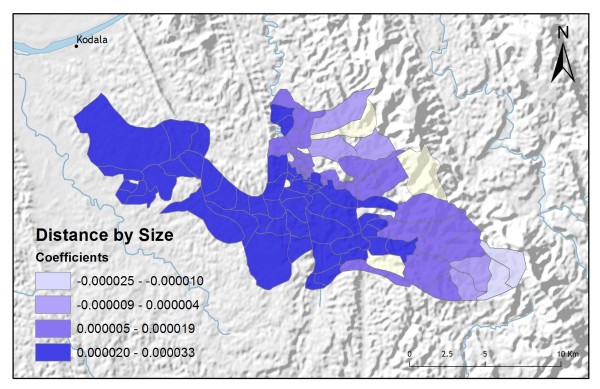
**Distance to regional centre by size GWR coefficients for predicting vendor service preference**.

**Figure 12:**
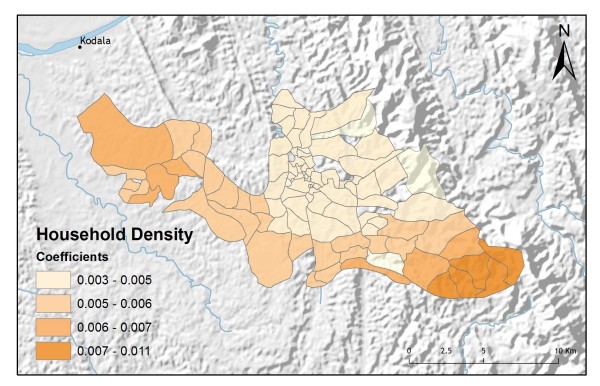
**Household density GWR coefficients for predicting control programme preference**.

**Figure 13:**
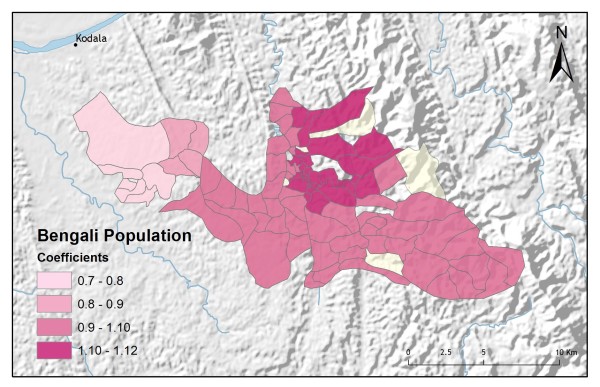
**Bengali population GWR coefficients for predicting control programme preference**.

**Figure 14:**
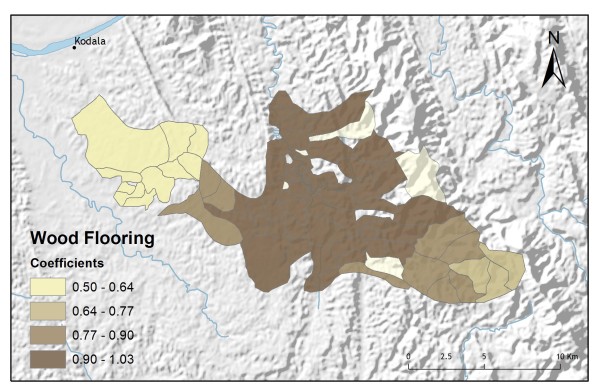
**Wood floor GWR coefficients for predicting control programme preference**.

In addition to improving model fit, GWR also provides useful information about explanatory variable stationarity [31]. Mapped coefficients for each village (Figures 10, 11, 12, 13 and 14) indicated where the explanatory variables were effective predictors of treatment preferences and where they were not. Education, for example, had a positive relationship to vendor use: as the number of people with no schooling increased, preference for vendor treatment also increased. In Figure 10, the villages rendered using the darkest colours indicate where the coefficient for the education variable is largest. The larger the coefficient is, the stronger the relationship is. The education variable is a strong predictor in the western-most villages only. Other variables exhibit this non-stationarity as well. The interaction term relating distance to the regional urban centre and village size also had a positive relationship with vendor use preference. Figure 11 shows that this explanatory variable is a very weak predictor in the eastern-most villages where the coefficient is near zero and even slightly negative; it is a strong predictor throughout the rest of the study area, however. Figure 12 looks at the coefficients for household densities found to be a key explanatory variable for predicting preference for control programme services. This variable predicts most strongly in the eastern- and also western-most villages; it is a less effective predictor in the central villages. Villages with larger numbers of Bengali people were also found to be associated with increased control programme preference. Figure 13 presents a map of the coefficients for that variable, indicating that the Bengali population is an especially good gauge of control programme preference in the northern central villages.

## Discussion

The models presented in this research indicate that a number of factors impact treatment-seeking practices in this region of Bangladesh. These factors include education, economics, village size in relation to remoteness, tribal affiliation, and household densities. The interaction term relating village size to remoteness had a positive relationship to vender use preference, and a negative relationship to control programme use preference. This suggested that larger villages, particularly those far from the area's primary regional hospital, have both the demand (people) and the motivation (accessibility issues) to support vendor services. While these villages do offer BRAC-provided control programme services, studies have shown that dropout rates among health workers is high due to low pay and in some cases, the number of health workers is insufficient [3,4]. There have also been cases where an individual with a fever uses control programme services, but when told they do not have malaria, they seek treatment from local vendors. Interviews with health volunteers in Kenya [34] indicate lack of trust in the community health workers was leading to alternative treatment-seeking strategies. These same issues may be impacting the health seeking behaviour in CHT area of Bangladesh. Other programmes that use community workers have increased their success by implementing tailored health worker training and support [35].

Previous studies [4] indicate the malaria control programme in the western villages of Rajasthali is being impeded by high vendor patronage. Since both the interaction variable and the variable representing the number of people with no formal education are strong predictors of vendor use preference in these areas, these villages are good candidates for educational programmes and/or advertising campaigns that warn against taking malaria medicines without proper diagnosis. Comprehensive research [36] on febrile illness along the Bangladesh-Myanmar border found only 40.9% of fever cases were *falciparum *malaria. Yet even with huge intervention efforts supported by the Global Fund Project, 48.8% of the people in this area prefer treatment from drug vendors without proper diagnosis [4]. A recent study from Uganda [37] discusses the feasibility of providing vendors with the rapid diagnostic test kits for malaria as a way to encourage proper diagnosis. This strategy could be effective for Bangladesh as well, where drug resistance is already a major hindrance to malaria control [38-40].

There is also a positive relationship between the number of homes constructed using wood flooring, and the number of people with control programme treatment preferences. Housing materials are a reflection of economic status. Wood and mud flooring are associated with poorer families. While the analyses were conducted at the village level and broad conclusions about individuals within villages is not appropriate (ecological fallacy), it may be that these families cannot afford vendor services. In that case, they would be more likely to take advantage of control programme services which are offered free of cost. There is also a strong association between malaria infection and housing materials [22,41-44]. An increase in malaria incidence for homes with wood flooring and/or poorer living conditions may play a role in promoting preferences for control programme services.

Another interesting correlation links larger communities of Bengali people with increased preference for control programme services. The Bengali communities represent populations that migrated from the plains and are well aware of their malaria risk. While there is no literature linking tribal affiliation with regard to treatment-seeking approaches in CHT, ethnic variations in treatment-seeking behaviour was noted in Bhutan [45].

While the Global Fund has contributed substantial resources towards malaria control in Bangladesh, more could be done to monitor progress and assess effectiveness. In particular, greater account should be given to local context, both cultural and environmental. An inspiration to other health education efforts, UNICEF [46] successfully collaborated with local community members to spread health messages. This example provides one piece of evidence that focusing on the social determinants of health and how they uniquely impact life in the CHT could lead to positive changes in malaria health practices.

This study illustrates that it is not enough for villages to provide local doctors and open office hours; offering services free of cost does not guarantee they will be fully utilized. It is essential to understand the factors that deter individuals from using these services, but it is also important to understand variations in how those services are provided across villages. Affordability, better economic support of health workers, acceptability, education, strong provider-patient trust, and availability have been shown to be key elements of a successful programme [47].

## Conclusion

Further research, incorporating both qualitative and quantitative methods, is needed to better understand why control programme resources are under-utilized. Discovering the villages at risk, and innovative ways to best communicate with them will ensure better diagnosis and treatment. Researchers have found that longer travel, waiting, and treatment times encourage people to use self-medication and promote the use of vendors [6,48-50]. Consequently, methods to improve access to malaria treatment in Bangladesh should be evaluated and operationalized. Since vendor usage remains high in the CHT, the government should formulate new policies to permit antimalarial drug sales by drug vendors. Developing appropriate tools for effective mass campaigning and awareness building among different communities is also needed to help change individual treatment-seeking approaches in the population. In this study, it was illustrated that, at a local level, treatment-seeking approaches for malaria treatment are highly heterogeneous. A study from Venezuela showed that a large reduction in malaria transmission using targeted control is feasible when the heterogeneity and spatial scale of malaria are correctly identified [51]. Therefore, mapping the risk of malaria based on fine-grained maps of villages and developing treatment behaviour maps could be of practical use in planning interventions in Bangladesh.

## Competing interests

The authors declare that they have no competing interests.

## Authors' contributions

UH and LS conceived the study design, prepared the data, performed the analysis, interpreted results and produced the final manuscript. MH, RH, TY and GG were responsible for the conception, overall scientific management, interpretation of results, and critically reviewed the final report. EF was responsible for the interpretation of results and critically reviewed the final report. Principal investigator of the project: UH. All authors read and approved the final manuscript.
